# A validation of the construct and reliability of an emotional intelligence scale applied to nursing students^1^

**DOI:** 10.1590/0104-1169.3498.2535

**Published:** 2015

**Authors:** Maritza Espinoza-Venegas, Olivia Sanhueza-Alvarado, Noé Ramírez-Elizondo, Katia Sáez-Carrillo

**Affiliations:** 2 PhD, Associate Professor, Departamento de Enfermería, Universidad de Concepción, Concepción, Chile; 3 PhD, Full Professor, Departamento de Enfermería, Universidad de Concepción, Concepción, Chile; 4 PhD, Professor, Universidad de Costa Rica, San José, Costa Rica; 5 PhD, Associate Professor, Departamento de Estadística, Facultad de Ciencias Físicas y Matemáticas, Universidad de Chile, Santiago, Chile

**Keywords:** Validity of Tests, Reproducibility of Results, Emotional Intelligence, Students, Nursing

## Abstract

**OBJECTIVE:**

The current study aimed to validate the construct and reliability of an emotional intelligence scale.

**METHOD:**

The Trait Meta-Mood Scale-24 was applied to 349 nursing students. The process included content validation, which involved expert reviews, pilot testing, measurements of reliability using Cronbach's alpha, and factor analysis to corroborate the validity of the theoretical model's construct.

**RESULTS:**

Adequate Cronbach coefficients were obtained for all three dimensions, and factor analysis confirmed the scale's dimensions (perception, comprehension, and regulation).

**CONCLUSION:**

The Trait Meta-Mood Scale is a reliable and valid tool to measure the emotional intelligence of nursing students. Its use allows for accurate determinations of individuals' abilities to interpret and manage emotions. At the same time, this new construct is of potential importance for measurements in nursing leadership; educational, organizational, and personal improvements; and the establishment of effective relationships with patients.

## Introduction

The concept of Emotional Intelligence (EI) surfaced several decades ago primarily through the work of Salovey and Mayer ^(^ 1 ^)^. As it became more popular, several models and tools for the measurement of EI emerged. In an effort to clarify this situation, the Encyclopedia of Applied Psychology suggested the existence of three major conceptual models^ (2)^: (a) the Bar-On model describes a transverse section of interrelated emotional and social competencies, abilities, and facilitators that influence intelligent behavior. Measurements for this model are obtained primarily through self-reports, the focus of which are potentially expandable and can include an interview and evaluation by multiple evaluators. (b) Goleman's model^ (3)^ understands EI through a wide range of competencies and abilities that increase work performance. (c) The model by Salovey-Mayer ^(^ 4 ^)^ defines EI as the ability to perceive, understand, manage, and regulate one's emotions, as well as the emotions of others.

This last model involves a multidimensional construct with three processes: perception, comprehension, and regulation of emotions. Perception involves the conscious recognition of one's emotions and one's ability to identify and verbally label what is being felt. Comprehension refers to the integration of what is thought and what is felt in addition to understanding how to consider the complexity of emotional changes. Finally, regulation refers to one's ability to efficiently direct and manage positive and negative emotions^ (1,4)^.

From this concept, Mayer and Salovey ^(^ 4 ^)^ designed one of the first measurement tools for EI, the 48-item version of the Trait Meta-Mood Scale (TMMS), which resulted from a systematic literature review of the essential factors for the emotional and social functioning of individuals. Its multidimensionality was confirmed using factor analysis, which demonstrated that perception, comprehension, and regulation (with Cronbach alpha scores of 0.86, 0.87, and 0.82, respectively) were three theoretical factors that could be measured using this scale^ (4)^. Prior studies have revealed excellent reliability and validity of results using shorter modified versions of the TMMS, which has been translated into several languages, including German^(^ 5 ^)^, Chinese^(^ 6 ^)^, Portuguese^(^ 7 ^)^, Persian^(^ 8 ^)^, Turkish^(^ 9 ^)^, and Basque^(^ 10 ^)^. A group of Spanish researchers^ (11)^ developed a valid adaptation of the TMMS that has been shown to have similar reliability and validity results of criterion and construct in successive applications in the Spanish population^ (12-13)^. After its development, this Spanish version has been used in several studies in Spanish-speaking Latin American countries^ (14-15)^, but not all authors report the validity and reliability levels obtained in their research. 

In Chile, there have been reports of using the EI scale in nursing students. However, the published studies only describe the reliability level. Although the reliability has been reported to be acceptable for each dimension (Perception 0.87; Comprehension 0.89; Regulation 0.85), this does not represent a pertinent analysis of the construct validity of the EI scale^ (15)^. Measuring this construct is especially relevant in the health field because the emotional dimension is a key factor in the area of personal interactions, where human care is manifested^ (16-17)^. Nursing is a profession that is particularly centered in this manner of care involving deep emotional connections. This specific characteristic of care leads nursing professionals to face complex situations that produce emotional reactions, such as high levels of anxiety, which they need to be able to manage effectively ^(^ 12 ^)^. Thus, the ability to manage one's emotions and to interpret the emotions of others is especially useful in the performance of nursing functions. Indeed, the capacity to evaluate and distinguish the emotional responses of patients can be decisive in the establishment of an efficient and significant relationship between the nursing professional and the individual receiving care^ (18)^. Likewise, several studies have shown the importance of acquiring emotional abilities because of the fact that strong emotional skills can promote professional leadership, better interdisciplinary team work, and improved work satisfaction^ (19)^. In contrast, poor management of emotional abilities can lead to professional deterioration and to worsening general health^ (20)^. 

It is particularly important for nursing students to begin learning how to interact with individuals in various health conditions, and they should thus be provided training in these abilities from the beginning of their educational program^ (21-22)^. Several studies that show the advantages and disadvantages of EI have reported that nursing students with better emotional abilities exhibit lower levels of anxiety regarding death^ (12,15,23)^ and a greater capacity for empathy^ (24)^. Other studies have reported that lower emotional abilities are associated with increased stress levels, especially when exposed to clinical learning^ (25)^, depressive behaviors, and low self-esteem ^(^ 26 ^)^. It has also been suggested^ (21)^ that EI could have a positive impact on the academic performance of students because it is significantly related to critical thinking. Thus, it is necessary for all nursing educational models to integrate and develop the emotional abilities of nursing students, especially in light of the research showing that it is possible to teach these abilities and that significant learning can be achieved after educational interventions^ (21)^. 

Based on this, the objective of this study is to validate the construct and validity of the Spanish version of the TMMS-24 EI Scale in a group of nursing students from two universities in Concepción, Chile. The purpose of this study is to validate the measurement elements in our culture and context to enable evaluations of the emotional competency of nursing students, which appears to be necessary for a better work and personal quality of life. 

## Methods

The psychometric evaluation process to assess validity and reliability was performed according to the literature recommendations^ (27)^. The first step in this process consisted of a review of content validity performed by experts in the area of psychology and nursing. These experts evaluated the conceptual equivalence or the degree to which the tool reflected the specific domain to be measured and the thematic cultural equivalence. Subsequently, a pilot test was administered to 30 students from another university. This pilot test demonstrated that the students understood the items and the scale, and it was thus not necessary to modify the scale.

Analyses of the test's reliability and construct validity were performed with the definitive sample, and these analyses took advantage of the fact that there were more than five elements per item of the scale^ (28)^. 

The reliability was examined by analyzing internal consistency using Cronbach's alpha reliability coefficient. The optimal range of alpha values is generally considered to be between 0.7 and 0.9^(^ 28 ^)^. The probability of error was set at 5%.

The validity of the EI construct was analyzed via confirmatory factor analysis, which made it possible to verify whether the factors and variables that made up the TMMS-24 scale were in congruence with the pre-established theory of tri-dimensionality^ (6)^. The stages described in the next section were followed for this analysis^ (28)^.

The correlation matrix between variables or scale items was created with the goal of reviewing the pattern of relationships between the variables (Pearson's r). From this, we also obtained a series of statistical tests that indicate if the factor analysis is pertinent with the available information. For example, the Kaiser-Meyer-Olkin (KMO) coefficient is considered to be adequate when it is greater than 0.6, and Bartlett's test of sphericity tests the null hypothesis that the variables are uncorrelated. A statistical significance level of less than 5% was accepted as valid.

The most widely used method of factor extraction is principal components analysis. This model generates as many factors as there are variables in the analysis. It first searches for the factor that can explain the largest amount of variance in the correlation matrix, which is subtracted from the original matrix. It then seeks a second linear combination that explains the maximum proportion of remaining variance, and so on. The extracted factors are not correlated amongst themselves. Factors with a variance greater than one must be incorporated; if not, it would explain a lower variance than an original variable.

Calculations of the similarities that measure the percentage of variance in a variable that is explained by all of the factors together can be interpreted as the reliability of the indicator.

Determining the number of factors to include in a model is typically an arbitrary decision. Different criteria have been defined, including the Kaiser criterion, which requires retaining all factors with a value greater than one. For the purpose of the current study, the Kaiser criterion was followed.

Factor rotations can facilitate the interpretation of extracted factors. The sum of the values is not affected by rotation, but rotation does alter the values and the percentage of explained variance. The Varimax method for matrix rotation was selected for this analysis.

An evaluation of model adjustment was performed, and the model was validated to determine the quality of the obtained factor solution. The resulting factors are interpreted, and they should contain all of the specific variables (or items) of each original dimension, thus allowing for the confirmation of previously proposed theoretical constructs. 

### Participants

The sample consisted of 349 nursing students (82% of the total population) from the first to fifth year in nursing programs at two different universities in the city of Concepción. The inclusion criteria considered all nursing students at the various levels of study from both universities and excluded those that were absent or on leave at the time of data acquisition. The majority were women (80%) between the ages of 17 and 37 years (x=21.3 s=2.7). Procedure: The questionnaires were administered before class, during which time the students were invited to participate and the study conditions were explained. The questionnaires were self-administered and took approximately 15 minutes to complete. Attendance was considered to be a random event. The responses from some students were omitted because of non-attendance (10%) or because they were incomplete (18%).

### Ethical aspects

Prior authorization to use the TMMS in this study was obtained from the authors of the Spanish version of the TMMS-24 scale. In addition, this study was approved by the Ethical Committee of the School of Medicine and the Nursing Departments of each university. Each student signed an informed consent form, which safeguarded their confidentiality and indicated the possibility of leaving the study at any time. Due to the connotation of the researched subject, the anonymity of the participating universities was preserved.

### Emotional Intelligence Tool

The EI Scale, originally called the Trait Meta-Mood Scale (TMMS-24) by Salovey and Mayer ^(^ 4 ^)^, which had been previously translated into Spanish,^ (11)^ measures EI. It consists of 24 items that are subdivided into three subscales or dimensions: emotional perception, emotional comprehension, and emotional regulation. The score for each of these subscales is classified into three ranges. For the emotional perception subscale, the middle score range (22 to 32 in men; 25 to 35 in women) indicates adequate emotional perception, and scores in the high (>33 in men; >36 in women) or low (<21 in men; <24 in women) range indicate that emotional perception should be improved. In contrast, for the comprehension subscale, scores in the low range indicate a need for improvement (<25 in men, <23 in women), those in the middle range (26 to 35 in men; 24 to 34 in women) indicate adequate comprehension, and those in the high range (>36 in men; >35 in women) indicate excellent emotional comprehension. Likewise, in the emotional regulation subscale, low scores (<23 in men and women) indicate the need for improvement, scores in the middle range (24 to 35 in men, and 24 to 34 in women) indicate adequate regulation, and high scores (>36 in men, >35 in women) indicate excellent emotional regulation. In the questionnaire, individuals must rate each of their responses on a Likert scale from one to five points to indicate their level of agreement. The total score is obtained by adding the responses from each sub-scale, each of which ranges from eight to 40 points.

## Results

Univariate analysis of the TMMS-24 scale demonstrates that the data are normally distributed. The scores obtained in all dimensions are within the range of adequate emotional perception, comprehension, and regulation, as shown in Table 1.

**Table 1 -:** Statistical measures of the TMMS-24 tool in nursing students, Concepción, Chile, 2012

Statistical measure	Perception Dimension	Comprehension Dimension	Regulation Dimension
Mean	27.8	27.21	29.87
Standard deviation	6.54	6.69	6.28
Asymmetry	0.13	0.13	0.13

n=349

### Reliability of the TMMS-24 scale

The internal reliability of the original TMMS was 0.95 (95%). Likewise, for each of its three dimensions, the Cronbach alpha values obtained were greater than 85%. Specifically, a Cronbach alpha of 88% was observed for the perception dimension, an alpha of 89% was observed for the comprehension dimension, and an alpha of 86% was observed for the regulation dimension. These results allow us to assert that the items are homogenous and that the scale consistently measures the characteristics for which it was created (Table 2).

**Table 2 -:** Statistical measures: total per item of the three dimensions of the TMMS-24 tool in nursing students, Concepción, Chile, 2012

Dimension		Mean of the scale if the item is eliminated	Variance of the scale if the item is eliminated	Corrected item/total correlation	Cronbach’s alpha if the item is eliminated
A) Perception Dimension*					
	1. Attention to feelings	23.65	35.72	0.53	0.87
	2. Concern for what is felt	23.89	33.04	0.74	0.85
	3. Takes time to think about emotions	24.26	32.77	0.71	0.86
	4. It is worthwhile to think about my emotions and mood	23.94	33.68	0.64	0.87
	5. I allow my feelings to affect my thoughts	24.91	35.34	0.43	0.89
	6. I constantly think about my mood	24.96	32.65	0.66	0.86
	7. I often think about my feelings	24.55	31.91	0.74	0.85
	8. I pay a lot of attention to how I feel	24.46	31.99	0.72	0.86
B) Comprehension dimension^†^					
	9. My feelings are clear to me	23.83	34.02	0.63	0.88
	10. I can frequently define my feelings	23.93	33.28	0.76	0.86
	11. I almost always know how I feel	23.85	33.88	0.74	0.87
	12. I know my feelings towards other people	23.62	36.14	0.61	0.88
	13. I know my feelings under different situations	23.56	35.81	0.68	0.87
	14. I can always say how I feel	24.03	33.72	0.69	0.87
	15. I can sometimes say what my emotions are	23.96	36.77	0.54	0.89
	16. I can come to understand my feelings	23.72	35.46	0.64	0.88
C) Regulation Dimension^‡^					
	17. Although I feel sad, I have an optimistic view	26.13	29.06	0.70	0.83
	18. Even if I don’t feel well, I try to have nice thoughts	26.13	28.65	0.79	0.82
	19. When I am very sad, I think about life’s pleasures	26.81	29.04	0.63	0.84
	20. I try to think positively even when I don’t feel well	26.29	28.59	0.77	0.82
	21. If I think too much about something I try to calm down	26.26	31.49	0.53	0.85
	22. I worry about being in a good mood	25.95	31.98	0.55	0.85
	23. I have a lot of energy when I feel happy	25.26	36.04	0.33	0.87
	24. When I am mad, I try to change my mood	26.24	31.54	0.53	0.85

* Cronbach's alpha, Perception dimension 0.88

† Cronbach's alpha, Comprehension dimension 0.89

‡ Cronbach's alpha, Regulation dimension 0.86

### Validity of the construct of the TMMS-24 scale

Table 3 shows a summary of the statistical measures of Pearson's correlation matrix for the items from the three sub-scales of the TMMS-24 prior to factor analysis. The correlation averages of the different scales were observed to be similar, although they are slightly higher in the emotional regulation sub-scale. The emotional regulation sub-scale also had the widest range of scores (3.05 to 4.60). The average correlation and the inter-element correlation both showed a moderately acceptable positive relationship for each of the sub-scales (r>0.4). The lowest correlation was observed for the emotional perception sub-scale.

**Table 3 -:** Summary of statistics of the correlation of items of the TMMS-24 sub-scales in nursing students, Concepción, Chile, 2012

		Mean	Minimum	Maximum	Range	Maximum/minimum	Variance	Items
Sub-Scale 1 – Emotional Perception								
	Mean of items	3.47	2.84	4.15	1.30	1.45	.22	8
	Variance of items	1.23	.954	1.426	.472	1.49	.02	8
	Inter-item correlation	.482	.246	.688	.442	2.797	.017	8
Sub-Scale 2 – Emotional Comprehension								
	Mean of items	3.40	3.18	3.65	.46	1.14	.02	8
	Variance of items	1.24	.99	1.56	.57	1.58	.03	8
	Inter-element correlation	.49	.27	.67	.39	2.41	.01	8
Sub-Scale 3 – Emotional Regulation								
	Mean of elements	3.73	3.05	4.60	1.54	1.50	.18	8
	Variance of elements	1.21	.52	1.65	1.12	3.12	.10	8
	Inter-element correlation	.42	.18	.72	.53	3.88	.02	8

**Table 4 -:** Matrix of rotated components of the TMMS-24 scale in nursing students, Concepción, Chile, 2012

Item	Component			
	1	2	3	h2
1. Attention to feelings		0.62		0.43
2. Concern for what I feel		0.81		0.68
3. Take time to think about my emotions		0.79		0.65
4. It is worthwhile to think about my emotions and mood		0.71		0.54
5. I let my feelings affect my thoughts		0.57		0.42
6. I think about my mood constantly		0.76		0.59
7. I often think about my feelings		0.83		0.69
8. I pay a lot of attention to how I feel		0.79		0.66
9. My feelings are clear to me	0.72			0.55
10. I can frequently define my feelings	0.82			0.70
11. I almost always know how I feel	0.79			0.67
12. I know my feelings towards others	0.72			0.54
13. I am aware of my feelings in different situations	0.73			0.59
14. I can always say how I feel	0.75			0.59
15. I can sometimes say what my emotions are	0.63			0.42
16. I can get to understand my feelings	0.68			0.53
17. Although I feel sad, I have an optimistic view			0.78	0.68
18. Although I feel poorly, I try to have nice thoughts			0.86	0.76
19. When I am very sad I think about life’s pleasures			0.74	0.57
20. I try to think positively even when I don’t feel well			0.85	0.74
21. If I think too much about things, I try to calm down			0.59	0.41
22. I worry about being in a good mood			0.59	0.49
23. I have a lot of energy when I am happy			0.35	0.23
24. When I am mad, I try to change my mood			0.62	0.42
Eigenvalues		4.71	4.68	4.16
56.5% Variance		19.6	19.5	17.3

Extraction method: Analysis of principal components Rotation method: Varimax normalization with Kaiser

Regarding the data in Table 2, it is possible to examine the contribution of each item that correlates with each sub-scale. The results demonstrate that each of the eight items is positively correlated within each sub-scale. Item 5 ("I allow my feelings to affect my thoughts") and item 23 ("I have a lot of energy when I feel happy") are the items that were observed to exhibit the lowest correlation with their respective sub-scale. Thus, the coefficients of reliability of the scale increase slightly when these items are eliminated. However, this variation does not substantially change the reliability of the TMMS, and these data do not justify eliminating these items to strengthen the validity of the scale.

From these results, we can assert that the items are homogenous and that the three sub-scales consistently measure the characteristics for which they were created, thus, they are reliable and demonstrate strong construct validity. 

### Factor analysis

The Kaiser-Meyer-Olkin test of appropriateness of the sample was 0.895, and the test of sphericity was significant (p<0.000), which allowed for a pertinent factor analysis. Saturations greater than 0.3 were gathered, and the criterion of using values greater than one was followed. The initial results prior to rotation identified three factors that accounted for 56.5% of the total variability of the data. To confirm the tri-dimensional hypothesis of the scale, which has been proposed, and to search for the best adjustment, the results of the Analysis of Principal Components were subjected to a Varimax rotation.

The results were similar and yielded a three factor structure. The first factor (explained variance = 19.6) grouped items one to eight and corresponds to the emotional perception dimension. The second factor (explained variance = 19.5) grouped items nine to 16 and corresponds to the emotional comprehension dimension. The third factor (explained variance = 17.3) grouped items 17 to 24 and corresponds to the emotional regulation dimension. Item 23 ("I have a lot of energy when I feel happy") had a saturation <0.4, but its factor weight was more significantly oriented towards factor three. In turn, this same item was observed to have the lowest ability to explain the variability within its respective sub-scale (23%). The variability of the remaining items ranged between 41% and 71% (Table 4). 

## Discussion

The findings of this study highlight the strong reliability and construct validity of the TMMS-24 scale of EI in a population of Chilean nursing students, thus confirming the theoretical model proposed by the authors^ (1,4)^.

With respect to the reliability of the EI scale, the results show that there is good internal consistency and homogeneity of the items. Specifically, this scale obtained a high level of reliability with Cronbach's alpha for each of the sub-scales, a finding that is similar to those reported previously in studies using the same tool in populations of nursing students, with Cronbach alpha values greater than 0.80 in all three dimensions^ (12,18)^. Similarly, a study of 451 Colombian adolescents obtained a Cronbach alpha for the TMMS-24 of 0.80 in perception, 0.76 in comprehension, and 0.75 in regulation^ (29)^. Likewise, the Portuguese version of the TMMS-24 applied to a sample of students obtained a Cronbach alpha of 0.80 for perception, 0.79 for comprehension, and 0.85 for regulation^ (7)^.

In this sense, we can emphasize that the reliability of the TMMS-24 is in accordance with the data obtained in other types of studies in which this scale was applied. In fact, the results are very similar to those reported by the creators of the TMMS-24 themselves, where the reported alpha values were between 0.86 and 0.90^ (8,11)^.

The statistical measures to validate the EI construct in each of the three dimensions originally proposed (i.e., emotional perception, comprehension, and regulation) reflect relationships that point in the same direction between the pairs of items. These analyses also show the contribution of each item to its respective sub-scale, thus confirming the pertinence of the variables or items proposed in the initial theoretical model^ (4)^.

Likewise, factor analysis contributes to the validation of the construct by demonstrating that the items tend to group themselves in the dimensions of perception, comprehension, and regulation, as initially proposed by the authors of the scale. In contrast to the first two dimensions, in which all eight of the corresponding items consistently group themselves, the third dimension includes item 23, which showed the lowest factor weight of all items. Nonetheless, its low variability within the scale did not significantly affect the good reliability levels or the originally proposed structure. Similar results have obtained by other researchers^ (13,30),^, one of whom even suggested eliminating item 23 from the scale ^(^ 30 ^)^ along with item five, which was also observed to have lower (but still acceptable) correlation levels than the rest of the items from its respective dimension in this study. In contrast, a different study^ (12)^ recommended keeping item 23 in the scale after verifying its effects in the model. 

Other studies that have examined the construct validity of the TMMS-24 using factor structure have reported differing results, but this could be due to sampling differences. For example, an Australian study^ (31)^ initially identified four factors, but eventually confirmed only three factors after further confirmatory analysis. A Turkish study^ (32)^ also confirmed the factor structure, but the item distribution differed from that in the original study.

## Conclusion

In conclusion, the tri-dimensionality and reliability of this scale are confirmed by the present results, which guarantee the quality of the tool in the studied population. 

Based on the presented data, we can establish that the application of the TMMS-24 scale measures EI. Because of this, it is a measurement that could be applied in additional populations of Chilean nursing students with the goal of greater generalization of the results. As seen in the evidence from other studies, this scale has been verified to show very similar results that confirm its theoretical framework. Thus, it may be used appropriately in nursing. More research, including investigations of its applicability and relevance for other health professions in Spanish-speaking countries, is suggested to potentially enhance the training of professionals.

In addition, this scale has characteristics, such as its ease of application, which enables gathering responses from a large number of surveyed individuals. Thus, this scale represents an easy and economical way to measure the EI construct.

However, we must consider the limitations of this study. The first is the fact that the questionnaire examined is a self-reporting tool in which individuals can respond based on their perceptions of socially desirable responses rather than their real "EI". The second limitation stems from the fact that the study sample was primarily composed of young females with similar levels of college education. Future studies should include a more heterogeneous sample.

## Relevance for nursing

This study establishes the contribution of a measurement tool that provides information on emotional management, the basis of EI. This new construct is of potential importance for enhancing nursing leadership and to facilitate educational, organizational, and personal improvements. In addition, this construct may help the establishment of an effective relationship with patients by interpreting and managing one's emotions, as well as the emotions of others, which could allow for improved adaptations in response to change, more effective resolutions of personal and interpersonal problems, and more efficient means of facing the daily demands, challenges, and pressures of nursing.

This tool has been used in several different cultural contexts, and good psychometric results have been observed in all of them. This characteristic reflects the strong transcultural validity of both the measurement tool and the EI theoretical model.

## References

[1] Salovey P, Mayer J.D (1990). Emotional intelligence. Imagination, Cognition, and Personality..

[2] Bar-On R (2006). The Bar-On model of emotional-social intelligence
(ESI).

[3] Goleman, D (1998). Working with emotional intelligence.

[4] Salovey P, Mayer J, Goldman S, Turvey C, Palfai T, Pennebaker James (1995). Emotional Attention, clarity, and repair: exploring
emotional intelligence using the trait meta-mood scale..

[5] Otto J, Döring-Seipel E, Grebe M, Lantermann ED (2001). Entwicklung eines Fragebogens zur Erfassung der
wahrgenommenen emotionalen Intelligenz Aufmerksamkeit auf, Klarheit und
Beeinflussbarkeit von Emotionen. Diagnostica..

[6] Li C, Yan J, Yin X, Wu Z (2002). A primary study of the application of the Trait
Meta-Mood Scale in military medical students. Chinese J Clin Psychol..

[7] Queirós MM, Fernández-Berrocal P, Extremera N, Cancela JM, Queirós PS (2005). Validação e fiabilidade da versão portuguesa modificada
da Trait Meta-Mood Scale. Rev Psicol Educ Cultura..

[8] Bayani AA (2009). Psychometric data for a Farsi translation of the Trait
Meta- Mood Scale. Psychol Reports..

[9] Aksöz I, Bugay A, Erdur-Baker Ö (2010). Turkish adaptation of the trait metamood
scale. Procedia Soc Behav Sci..

[10] Gorostiaga A, Balluerka N, Haranburu M, Alonso-Arbiol I (2011). Measuring perceived emotional intelligence in adolescent
population: validation of the Short Trait Meta-Mood Scale
(TMMS-23). Int J Clin Health Psychol..

[11] Fernandez -Berrocal P, Extremera N, Ramos N (2004). Validy and reliability of the Spanish modified version
of the Trait Meta- Mood Scale. Psycol Rep..

[12] Aradilla-Herrero A, Tomás-Sábado J, Gómez-Benito J (2013). Perceived emotional intelligence in nursing:
psychometric properties of the Trait Meta-Mood Scale. J Clin Nurs..

[13] Salguero JM, Fernandez-Berrocal P, Balluerka N, Aritzeta A (2010). Measuring perceived emotional intelligence in the
adolescent population: psychometric properties of the Trait Meta-Mood
Scale. Soc Behav Personal..

[14] Perdomo C, Pérez DM, Ibañez I (2011). Inteligencia emocional en adolescentes de dos colegios
de Bogotá y variables asociadas. Rev Colomb. Psiquiatr..

[15] Espinoza M, Sanhueza O (2012). Miedo a la muerte y su relación con la inteligencia
emocional de estudiantes de enfermería de Concepción. Acta Paul Enferm..

[16] Bulmer-Smith K, Profetto-McGrath J, Cummings GG (2009). Emotional intelligence and nursing: an integrative
literature review. Int J Nurs Studies..

[17] Chew BH, Zain AM, Hassan F (2013). Emotional intelligence and academic performance in first
and final year medical students: a cross-sectional study. BMC Med Educ..

[18] Aradilla-Herrero A, Tomás-Sábado J, Wergers CE (2011). The role of emotional intelligence in
nursing. Nursing Students and Their Concerns.

[19] Akerjordet K, Severinsson E (2010). The state of the science of emotional intelligence
related to nursing leadership: an integrative review. J Nurs Manage..

[20] Sharif F, Rezaie S, Keshavarzi S, Mansoori P, Ghadakpoor S (2013). Teaching emotional intelligence to intensive care unit
nurses and their general health: a randomized clinical trial. Int J Occup Environ Med..

[21] Fernandez R, Salamonson Y, Griffiths R (2012). Emotional intelligence as a predictor of academic
performance in first-year accelerated graduate entry nursing
students. J Clin Nurs..

[22] Sanjuán Quiles A, Ferrer Hernández M (2008). Perfil emocional de los estudiantes em prácticas
clínicas. Acción tutorial em enfermería para apoyo, forma ción, desarrollo y
control de las emociones. Invest Educ Enferm..

[23] Aradilla-Herrero A, Tomas-Sabado J, Gomez-Benito J (2013). Death attitudes and emotional intelligence in nursing
students. Omega: J Death Dying..

[24] Aguilar M, Augusto JM (2009). Relación entre inteligencia emocional percibida,
personalidad y capacidad empática en estudiantes de enfermería. Psicol Conduct..

[25] Chan MF, Creedy DK, Chua TL, Lim CC (2011). Exploring the psychological health related profile of
nursing students in Singapore: a cluster analysis. J Clin Nurs..

[26] Aradilla-Herrero A, Tomás-Sábado J, Gómez-Benito J (2014). Associations between emotional intelligence, depression
and suicide risk in nursing students. Nurse Educ Today..

[27] Reichenheim M, Moraes C (2007). Operacionalização de adaptação transcultural de
instrumentos de aferição usados em epidemiologia. Rev Saúde Pública..

[28] Meliá JL (1990). Construcción de la psicometría como ciencia teórica y
aplicada.

[29] Perdomo C, Pérez DM, Ibañez I (2011). Inteligencia emocional en adolescentes de dos colegios
de Bogotá y variables asociadas. Rev Colomb Psiquiatr..

[30] Martın-Albo J, Nuñez JL, Leon J (2010). Analysis of the psychometric properties of the Spanish
version of the Trait Meta-Mood Scale in a sports context. Psychol Reports..

[31] Palmer B, Donaldson C, Stough C (2003). Examining the structure of the Trait Meta-Mood
Scale. Austr J Psychol..

[32] Aksöz I, Bugay A, Erdur-Baker Ö (2010). Turkish adaptation of the trait metamood
scale. Procedia Soc Behav Sci..

